# Convergent construct validity and test-retest reliability of both German versions of the original and the revised Niigata PPPD Questionnaire: NPQ and NPQ-R

**DOI:** 10.3389/fneur.2025.1517566

**Published:** 2025-01-27

**Authors:** Sarah Chételat, Eve-Yaël Gerber, Sarah El Khadlaoui, Frank Behrendt, Michaela Stark, Stefan Schädler, Maximilian Maywald, Lena Fabritius, Johannes Gerb, Denis Grabova, Wiebke Trost, Andreas Zwergal, Ralf Strobl, Katrin Parmar, Hans Ulrich Gerth, Leo H. Bonati, Sandra Becker-Bense, Corina Schuster-Amft

**Affiliations:** ^1^School of Health Professions, Institute of Physiotherapy, Zurich University of Applied Sciences, Winterthur, Switzerland; ^2^Department of Research, Reha Rheinfelden, Rheinfelden, Switzerland; ^3^Department of Psychology, University of Basel, Basel, Switzerland; ^4^German Center for Vertigo and Balance Disorders (DSGZ), University Hospital, LMU Munich, Munich, Germany; ^5^School of Engineering and Computer Science, Bern University of Applied Sciences, Biel, Switzerland; ^6^Physiotherapie Stefan Schädler, Sumiswald, Switzerland; ^7^Department of Psychiatry and Psychotherapy, University Hospital, LMU Munich, Munich, Germany; ^8^Department of Neurology, University Hospital, LMU Munich, Munich, Germany; ^9^Institute for Medical Information Processing, Biometry, and Epidemiology (IBE), LMU Munich, Munich, Germany; ^10^Departments of Head, Spine and Neuromedicine and Biomedical Engineering, Translational Imaging in Neurology (ThINk) Basel, University Hospital Basel and University of Basel, Basel, Switzerland; ^11^Department of Medicine, University Hospital Münster, Münster, Germany; ^12^Department of Neurology, Stroke Center, University Hospital Basel, Basel, Switzerland; ^13^Department of Clinical Research, University of Basel, Basel, Switzerland; ^14^Department of Sport, Exercise and Health, University of Basel, Basel, Switzerland

**Keywords:** Persistent Postural-Perceptual Dizziness, functional dizziness, patient-reported outcome measure, concurrent construct validity, test-retest reliability, internal consistency, standard error of measurement, minimal detectable change

## Abstract

**Background:**

Persistent Postural-Perceptual Dizziness (PPPD) is a frequent chronic functional disorder that manifests with dizziness, unsteadiness, or non-spinning vertigo present for at least 3 months. Characteristic provocation factors are moving or complex visual stimuli and exclusion of organic diseases. To assess the severity and impact of PPPD, Japanese researchers developed the Niigata PPPD Questionnaire (NPQ). The study's aim was to evaluate the concurrent construct validity and reliability [including test-retest reliability, internal consistency, standard error of measurement (SEM), and minimal detectable change (MDC)] of the German version of the NPQ (12 items) and its revised version, NPQ-R, which contains 19 items addressing additional symptoms and symptom behavior.

**Methods:**

The Swiss Reha Rheinfelden and the German Center for Vertigo and Balance Disorders included 265 PPPD patients (mean age 50.2 ± 16.8 years, disease duration 46.3 ± 76.6 months). Patients completed the NPQ and the NPQ-R (twice), the DHI and potentially related constructs: anxiety (ABC-Scale, VSS), depression (HADS), and general health (SF-36) once. To assess the questionnaires' reliability and validity, several statistical measures were calculated, including Spearman's rank correlation coefficients, Intraclass Correlation Coefficients (ICC_2, 1_), Cronbach's alpha, SEM, and MDC.

**Results:**

On average, patients scored 29.9 ± 13.2 for NPQ and 52.3 ± 19.6 for NPQ-R. Correlations between NPQ/NPQ-R and (1) disease-specific questionnaires were r_s_= 0.712 and r_s_= 0.752 (DHI), r_s_=0.426 and r_s_= 0.0.462 (VSS-V), r_s_= -0.500 and r_s_= -0.545 (ABC-Scale), (2) anxiety-specific subscales rs = 0.394 and rs = 0.430 (VSS-A) and r_s_= 0.354 and r_s_= 0.430 (HADS-A), (3) depression-related subscales r_s_=0.438 and r_s_= 0.487 (HADS-D), and (4) general health r_s_ ranged between r_s_= -0.216 and −0.578 (all SF-36 subscales). Internal consistency, test-retest reliability, SEM and MDC calculated for NPQ/NPQ-R were α = 0.88/α = 0.91, ICC=0.83 (CI 0.77 to 0.0.87), SEM 5.55/8.37, and MDC 15/23 points.

**Conclusion:**

The German versions of NPQ and NPQ-R are valid and reliable patient-reported outcome measures for assessing PPPD, demonstrating satisfactory psychometric measurement properties including convergent construct validity and reliability parameters: internal consistency, test-retest reliability, SEM, and MDC as an evaluative measure. The NPQ-R, with its additional subscales addressing associated symptoms and symptom behavior, represents both the patient and clinician perspective on PPPD-specific problems. Therefore, we recommend utilizing the NPQ-R for a comprehensive assessment of PPPD.

## 1 Background

Persistent Postural-Perceptual Dizziness (PPPD) is the current term for chronic vertigo and dizziness syndromes characterized by one or more vestibular symptoms, including vertigo, dizziness, unsteadiness, or imbalance in stance and gait, in the absence of an identifiable organic etiology (1, 2). Functional dizziness syndromes have been recognized since the latter half of the 19th century and are currently the most prevalent types of vestibular disorders in our society, particular within specialized vertigo centers (up to 20%) (1–3). However, it was not until 2017 that the committee for the International Classification of Vestibular Disorders (ICVD) of the Bárány Society, the International Society of Neurootology, developed the first consensus document defining functional vertigo and dizziness (Table 1). This document introduced the new term “Persistent Postural-Perceptual Dizziness” (PPPD) along with its classification criteria (1). PPPD can develop following an acute organic vestibular or non-vestibular disorder, resulting in secondary PPPD (sPPPD), or it can develop independently in the absence of somatic triggers, referred to as primary PPPD (pPPPD) (1, 4). Its core features are based on common findings in syndromes described earlier such as “phobic postural vertigo”, “space-motion discomfort”, “functional vertigo”, “visual vertigo”, or “chronic subjective dizziness” (1, 2).

**Table 1:** Diagnosis criteria of persistent postural-perceptual dizziness (PPPD) from the committee for the Classification of Vestibular Disorders of the Bárány Society (according to Staab et al., (1), p. 196).

**Criteria for PPPD diagnosis**	
A	One or more symptoms of dizziness, unsteadiness, or non-spinning vertigo are present on most days for 3 months or more. 1. Symptoms last for prolonged (hours-long) periods of time, but may wax and wane in severity. 2. Symptoms need not be present continuously throughout the entire day.
B	Persistent symptoms occur without specific provocation, but are exacerbated by three factors: 1. Upright posture, 2. Active or passive motion without regard to direction or position, and 3. Exposure to moving visual stimuli or complex visual patterns
C	The disorder is precipitated by conditions that cause vertigo, unsteadiness, dizziness, or problems with balance including acute, episodic, or chronic vestibular syndromes, other neurologic or medical illnesses, or psychological distress. 1. When the precipitant is an acute or episodic condition, symptoms settle into the pattern of criterion A as the precipitant resolves, but they may occur intermittently at first, and then consolidate into a persistent course. 2. When the precipitant is a chronic syndrome, symptoms may develop slowly at first and worsen gradually.
D	Symptoms cause significant distress or functional impairment.
E	Symptoms are not better accounted for by another disease or disorder.

To date, there is no valid measurement instrument available in German-speaking countries for the assessment of PPPD-specific subjective patients' complaints and their severity (1, 5). The currently available patient reported outcome measurement tools, such as the Dizziness Handicap Inventory (DHI) or the Vertigo Symptom Scale (VSS), primarily address general dizziness issues. However, they do not adequately capture the specific complaints reported by patients with PPPD who do not exhibit an organic vestibular deficit. Such specific symptoms would particularly encompass the characteristic situational exacerbation factors associated with PPPD, including maintaining an upright posture, engaging in active or passive movement, and exposure to dynamic or complex stimuli (6, 7).

Based on the relatively new diagnostic criteria of the Bárány Society, Yagi and colleagues developed the first PPPD-specific patient-reported outcome measure in 2019: the Niigata PPPD Questionnaire in Japanese (NPQ) (8). The NPQ consists of 12 items divided into three subscales that evaluate the patient's dizziness complaints. The subscales focus on three typical PPPD provocation and/or amplication factors in PPPD: (1) being in an upright posture or while walking, (2) during movement, and (3) during visual stimulation. Twelve questions regarding these three reinforcing factors are evaluated using a Likert scale ranging from zero (no impact) to six (indicating unbearable impact), resulting in a total possible score of 72 points. In a retrospective study involving 50 patients with PPPD and 50 individuals with various vestibular syndromes, such as Menière's disease, Yagi and colleagues investigated the questionnaire's capacity to discriminate between the two patient groups (discriminate validity). It resulted in an initial valid and reliable patient-reported outcome measurement tool. The visual stimulation scale proved to be the most suitable for discrimination (8). However, the original NPQ exhibits some methodological flaws: (1) the items address only certain aspects of the diagnostic criteria, notably omitting considerations of psychosocial functioning impairment; (2) more attention could have been placed on scale's development and validation, particularly in accordance with the COSMIN guidelines that recommend the inclusion of both patient and expert perspectives during the development process of a patient-reported outcome measure focusing on content validity; and (3) a larger sample size for validation purposes is required (9).

Due to these shortcomings and to facilitate the accessibility of this measurement instrument in German-speaking countries, an official German translation of the NPQ was created including forward and backward translation by three officially recognized translators and the study team after consultation with the authors of the NPQ, based on existing guidelines (10). Additionally, the perspective from 28 health professionals specializing the field of dizziness and vertigo, as well as 11 patients diagnosed with PPPD, were included based on a three-round Delphi procedure and semi-structured interviews, respectively (11). The modified NPQ-R resulted in the addition of seven new items (now 19 items) and two new subscales (now five subscales). These modifications included additional questions addressing symptom behavior and psychosocial impairment associated with PPPD symptoms, such as difficulties with concentration. The NPQ-R now provides a comprehensive PROM for patients with PPPD-related symptoms that can comprehensively capture their symptoms. The process of expansion and all content-related details are described in Behrendt et al. (11). The German and the English versions of the NPQ and the NPQ-R are provided as Supplementary files 1–4. The evaluation scale remained the same Likert scale as in the original Japanese NPQ from zero (none) to six (6 = unbearable). The new NPQ-R total score can range from zero to 114 points.

The aim of the present study was to examine convergent construct validity and test-retest reliability of the German version of the Niigata PPPD Questionnaire (NPQ) and its revised version (NPQ-R) to support diagnostic procedures for PPPD and the monitoring of the treatment progress. We hypothesized a strong correlation r > 0.5 between the total scores of the NPQ/NPQ-R and the total scores of the DHI and VSS, as all three instruments assess vestibular disease-specific constructs.

## 2 Methods

### 2.1 Study design

This prospective study, comprising two measurement events, was designed to evaluate the internal consistency, test-retest reliability, standard error of measurement and convergent construct validity of the German NPQ and NPQ-R. The study was conducted in accordance with the Helsinki Declaration and received approval from the Ethics Committee Northwest and Central Switzerland (reference number: 2021-00974) as well as the Institutional Review Board of the Ludwig-Maximilians-University Munich Germany (reference number: 23-0126). All patients were provided with written and oral information about the project following the initial contact and were subsequently invited to participate in the study. Written informed consent was obtained from all participants prior to the start of data collection.

### 2.2 Participants

Patient inclusion criteria were diagnosis of PPPD, age > 18 years, good knowledge of German, and a signed informed consent. The exclusion criteria included the presence of other forms of vertigo.

In this bi-center study, patients from Switzerland and Germany were included. In Switzerland, outpatients were recruited from PPPD-specialists from the Oto-Rhino-Laryngology Clinics of the Cantonal Hospitals of Lucerne, the University Hospitals Zurich and Basel, the Reha Rheinfelden, and private practices. In Germany, all patients were recruited through the outpatient unit of the German Center for Vertigo and Balance Disorders (DSGZ) at the LMU University Hospital in Munich. PPPD diagnosis was confirmed according to the criteria established by the Bárány Society (1). The routine clinical assessment in all patients comprised a comprehensive clinical neurological, neuro-orthoptic, and neuro-otological examination, which encompassed procedures such as bithermal caloric irrigation and video head impulse test to evaluate both low- and high-frequency ranges of the vestibulo-ocular reflex. Neuro-orthoptic examination included detailed ocular motor examination, measurements of the subjective visual vertical and fundusphotography by use of a laser scanning opthalmoscope. The latter was especially important in order to exclude acute vestibular tone imbalance, e.g., spontaneous or head shaking nystagmus, skew deviation, head tilt, tilts of the subjective visual vertical or cyclorotation of the eyes (ocular torsion) as parts of the so-called ocular tilt reaction (12, 13). If useful, additional examinations such as static posturography, gait analysis, pure tone audiometry, or vestibular-evoked myogenic potentials were performed in a standardized manner. Patients with secondary PPPD with a residual unilateral peripheral deficit in the high- and/or low frequency range of the vestibulo-ocular reflex following a preceding acute unilateral vestibulopathy were included in the study only if this deficit was completely compensated centrally. Specifically, this meant that patients demonstrated no signs of acute vestibular tone imbalance and exhibited no vestibular sway patterns during static posturography. Additionally, the German patients were classified as either primary (p) or secondary sPPPD depending on the presence or absence of a confirmed preceding somatic trigger.

### 2.3 Measures

The NPQ and NPQ-R are utilized to assess the patient's subjective perception of severity, impairments, and limitations due to PPPD, thereby functioning as evaluative instruments (11). To assess the construct validity of the German versions of NPQ and NPQ-R, patients were asked to complete the following five patient-reported outcome measures:

1) The Dizziness Handicap Inventory (DHI) is a disease specific questionnaire assessing the impact of dizziness including 25 items within three subscales: physical, emotional and functional (14). The DHI evaluates items on an ordinal scale, which consists of the response options “yes” (four points), “sometimes” (two points) or “no” (zero points). The total score ranges from zero to 100, with the expression from “no disability” to “strongest perceived disability”. Overall, the German version of the DHI shows moderate to good psychometric properties (α = 0.90, ICC_2/1_ = 0.95 (CI 0.91, 0.98), concurrent validity: r = −0.53 to −0.72 with the SF-36, r = −0.64 with Activities-specific Balance Confidence (ABC) Scale, r = 0.41 with Hospital Anxiety and Depression Scale, subscale anxiety (HADS-A), r = 0.66 with Hospital Anxiety and Depression Scale, subscale depression (HADS-D) (7, 15).
2) The SF-36 Health Survey (SF-36) is a generic assessment tool measuring the perceived health related quality of life. The questionnaire contains 36 items comprising eight subscales and two composite domains that reflect physical and mental health. The subscales indicate physical functioning (pfi), role limitations due to physical problems (rolph), physical pain (pain), general health perceptions (ghp), vitality (vital), social functioning (soc), role limitations due to emotional problems (rolem), general mental health (mhi). Scoring is conducted by a computerized evaluation program by summing up the item responses per subscale whereby special weightings are applied for some subscales. The German Version of the SF-36 showed satisfactory psychometric properties, in particular for people with dizziness: internal consistency: α = 0.72–0.89, reliability ICC: 0.79 to 0.97, concurrent validity: r = −0.53 to −0.72 with the DHI.

PPPD has often been associated with psychiatric comorbidities, in particular anxiety and depressive disorders (16–18). However, PPPD is considered a functional diagnosis rather than a psychiatric diagnosis (19). To evaluate the association between PPPD symptoms and anxiety and depressive disorders, the following patient-reported outcome measures were used:

3) The German Version of the Vertigo Symptom Scale (VSS-G) (6, 20) evaluates the frequency and severity of dizziness symptoms within the last 12 months. It is composed of 34 items categorized into two subscales: autonomic arousal and somatic anxiety (VSS-A), and vertigo and related symptoms (VSS-V). This questionnaire is scaled to ordinal level, with response options reflecting frequency: zero (never), once to three times per year (few times, one point), four to twelve times per year (several times, two points), on average > 1/month (quite often, three points), and on average > 1/week (very often, four points) resulting in a total score range between zero and 136 points, indicating a spectrum from no symptoms to severe vertigo. The psychometric properties of the VSS-G scale show moderate to good internal consistency: α = 0.90, reliability: ICC = 0.93, and convergent validity: VSS-G: r = 0.56 with DHI, VSS-A: r = 0.45 with HADS-A and for discrimination VSS-V: r = 0.19 with HADS-A (6, 20).
4) The ABC scale is used as an instrument to assess functional mobility impairment (21). Comprising 16 items, the questionnaire assesses individuals' confidence in maintaining their balance during various daily activities, such as standing on a chair. The ABC scale ranges from zero (not at all confident) to 100 percent (absolutely confident) (21). Scores below 50 percent indicate a low level of physical functioning, while scores between 50 and 80 percent indicate a moderate level of physical functioning. Healthy, physiically active adults achieve scores above 80 percent (22, 23). The German Version of the ABC Scale (ABC-D) demonstrated satisfactory to very good psychometric properties (internal consistency: α = 0.91–0.95, reliability: ICC = 0.94, criterion validity: r = 0.57 with the SF- 36 domain physical health, r = 0.59 with the SF-36 domain mental health (22, 23).
5) The HADS is a concise self-report questionnaire designed for individuals with physical impairments, assessing levels of anxiety and depression in relation to the preceding week (15, 24). The 14-item questionnaire consists of two subscales: anxiety (HADS-A) and depression (HADS-D). Four ordinal response categories include zero (not at all), one (not very much/sometimes), two (quite a lot/very often), and three points (very much indeed/nearly all the time) (24). The score for each subscale ranges from zero to 21 points. A score ≥ eight defines the caseness on both subscales (25). Scores per subscale ≥eleven indicate severe to very severe symptomatology. The questionnaire has demonstrated good psychometric properties (internal consistency: α = 0.8 to 0.81), reliability: ICC = 0.81 to 0.89, construct validity HADS-A: r = 0.65 with other anxiety rating procedures, HADS-D r = 0.7 with other depression rating procedures, in different patient populations, e.g., neurologic or psychosomatic patients (25–27).

Furthermore, all patients completed a form to collect demographic information for descriptive purposes. Additionally, a subsample of the patients in Switzerland received an assessment sheet to evaluate the comprehensiveness and comprehensibility of the NPQ-R.

### 2.4 Procedures

**Figure 1:**
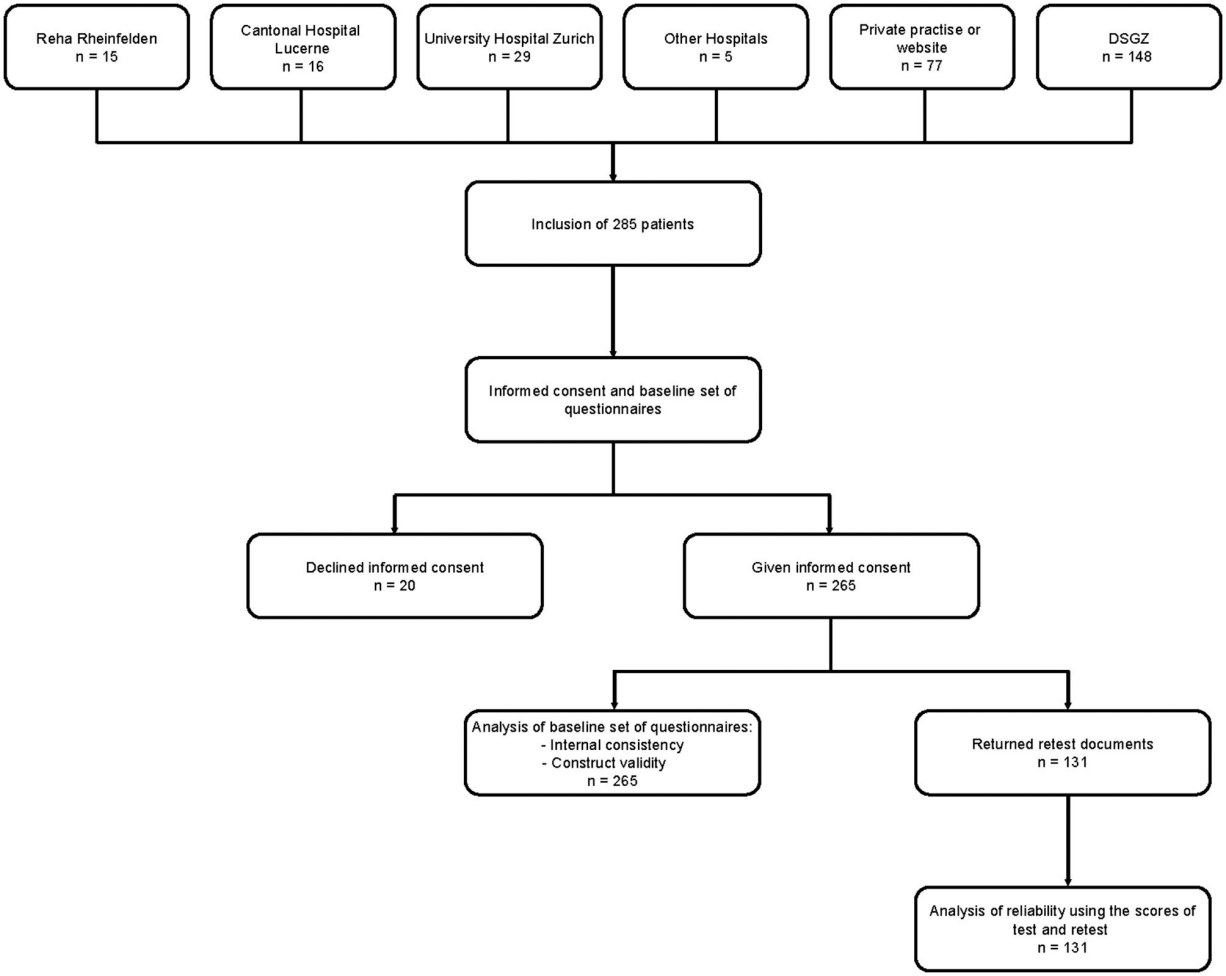
Flow Chart of data collection and analysis procedure. DSGZ, German Center for Vertigo and Balance Disorders; n, number of participants.

At baseline, all participants received the aforementioned questionnaires either by postal mail or through personal contact. The questionnaires were accompanied by an informational letter detailing the procedure and a stamped, addressed envelope for return. The procedure is illustrated in Figure 1.

In a subset of patients, the NPQ-R which includes the NPQ, was subsequently collected a second time at least 6 days later by postal mail. Additionally, the collection and processing of the responses were monitored weekly, including reminders sent by email or phone for any unanswered questionnaires or unclear responses.

### 2.5 Statistical analysis

Questionnaires with missing values exceeding 15% were excluded from analysis to mitigate potential selection bias (28). Demographics and test data were analyzed using descriptive statistics while frequency distributions were described using histograms. According to Terwee et al., floor or ceiling effects are present when 15% of subjects achieve the highest or lowest possible score (29).

In order to **pool the data** from Germany and Switzerland, NPQ-R and NPQ-R-Retest were tested for equivalence using an Independent Sample T-Test. The Equivalence Independent Samples T-Test results indicated that there are no statistically significant differences in both the total NPQ-R and NPQ-R-Retest scores, as evidenced by *p* > 0.05, specifically 0.886 and 0.231, respectively. Additionally, the equivalence bounds for Cohen's d were within the range of −0.003 to 0.003 for the NPQ-R and −0.002 to 0.002 for the NPQ-R-Retest, further suggesting no significant differences. Normal distribution of NPQ-R and NPQ retest scores were evaluated by visual inspection and the Shapiro-Wilk Test. The Shapiro-Wilk test results for Reha Rheinfelden indicate that both NPQ-R and NPQ-R Retest follow a normal distribution, with *p*-values of 0.704 and 0.730, respectively. Similarly, for DSGZ, both NPQ-R and NPQ-R Retest showed normal distribution, with *p*-values of 0.763 and 0.304. In order to get an overview of the **scale structure**, Cronbach's α was calculated to determine internal consistency whereby values between 0.7 and 0.95 are considered acceptable (30). Additionally, the comparisons to the original NPQ serves as a reference.

To determine **reproducibility of the instrument**, test-retest reliability was examined using intraclass correlation coefficients (ICC; two-way mixed effect model, absolute agreement between single scores) (31, 32). ICC values ≥0.70 are considered as the minimum standard for reliability in a study population of at least 50 subjects (29).

To assess not only the variability (reliability parameter) between the participants, but also the **agreement between repeated measurements** (agreement parameter), the measurement error (standard error of measurement, SEM) and the limits of agreements were also calculated. The minimal detectable change (MDC) is based on the measurement error (28).

To evaluate **convergent construct validity**, the scores of the NPQ-R were correlated with the scores of the DHI, VSS, ABC Scale, HADS, and SF-36. The correlation of the ordinal scaled variables was calculated using the Spearman's rank correlation coefficient (33) with guidelines proposed by Cohen: r_s_ = 0.10 to 0.29 = low correlation; r_s_ = 0.30 to 0.49 = medium/moderate correlation; r_s_ = 0.50 to 1.0 = large/high correlation (34). Regarding construct validity, we hypothesized that a high correlation r > 0.5 according to Cohen (34) will exist between the total score of the NPQ-R and the total score of the DHI and the VSS because all three instruments are related as disease-specific patient reported outcome measures. The NPQ and NPQ-R scores were statistically compared in primary PPPD vs. PPPD by *t*-test.

Level of significance was set at *p* ≤ 0.05. Statistical analyses were performed with R version 2021.09.2 (35), SPSS version 22.0.0.0 (IBM Corp. Armonk, US), and JASP version 0.16.2 (University of Amsterdam, The Netherlands).

## 3 Results

### 3.1 Study sample and setting

The study included 265 patients (130 males and 135 females). One hundred seventeen patients were recruited from different centers in Switzerland (between September 2021 and December 2023) and 148 patients were recruited from the DSGZ at the Ludwig-Maximilians-University Munich (GER, between April 2023 and December 2023). Descriptive data of all 265 patients for the two study centers are summarized in Table 2. After ensuring equivalence, data from both study centers were merged into a single master data file for all subsequent analyses.

**Table 2:** Descriptive analysis of both recruiting centers.

	Reha Rheinfelden/Switzerland (*n* = 117)^*^	LMU DSGZ/Germany (*n* = 148)^*^
Age	54.7 ± 18.3 (17.0/83.0)	46.6 ± 14.5 (19.0/74.0)
Gender	Female = 64	Female = 71
	Male = 53	Male = 77
Dizziness duration [months]	*n =* 102: 59.4 ± 87.6 (2.0/432.0)	37.2 ± 66.9 (1.0/401.0)
Duration of therapy [months]	*n =* 86: 8.6 ± 17.7 (0.0/144.0)	*n =* 147: 3.2 ± 9.4 (0.0/60.0)
Sport/week [number of times]	*n =* 108: 2.5 ± 2.2 (0.0/7.0)	2.4 ± 2.2 (0.0/7.0)
NPQ (12 items) [= to 72 points]	30.3 ± 13.4 (0.0/63.0)	29.5 ± 13.1 (2.0/59.0)
NPQ-R (19 items) [= to 114 points]	52.5 ± 20.0 (4.0/100.0)	52.2 ± 19.3 (4.0/93.0)
NPQ-R-Retest [= to 114 points]	*n =* 115: 49.4 ± 20.7 (2.0/102.0)	*n =* 16, 42.9 ± 22.6 (9.0/77.0)
DHI [0–100 points]	46.7 ± 20.2 (0.0/82.00)	45.66 ± 19.2 (8.0/88.0)
VSS-Total [0–136 points]	44.1 ± 25.4 (0.0/113.00)	41.39 ± 21.3 (4.0/96.0)
VSS-V [0–76 points]	21.5 ± 16.0 (0.0/65.00)	20.67 ± 13.6 (0.0/57.0)
VSS-A [0–60 points]	22.5 ± 12.5 (0.0/51.0)	20.7 ± 10.9 (1.0/50.0)
ABC [0–100%]	0.7 ± 0.2 (0.05/1.0)	*n =* 147: 0.7 ± 0.2 (0.01/1.0)
HADS-A [0–21 points]	7.7 ± 4.3 (0.0/20.0)	*n =* 147: 8.3 ± 4.5 (0.0/19.0)
HADS-D [0–21 points]	6.2 ± 4.0 (0.0/19.0)	*n =* 147: 6.8 ± 4.2 (0.0/19.0)
SF36.pfi	*n =* 116: 68.5 ± 25.8 (10.0/100.0)	*n =* 147: 69.9 ± 24.3 (0.0/100.0)
SF36.rolph	*n =* 114: 46.9 ± 42.2 (0.0/100.0)	*n =* 146: 39.6 ± 41.2 (0.0/100.0)
SF36.pain	*n =* 114: 61.91 ± 28.3 (0.0/100.0)	*n =* 146: 64.8 ± 28.7 (12.0/100.0)
SF36.ghp	*n =* 115: 54.0 ± 19.7 (5.0/97.0)	*n =* 145: 51.9 ± 20.9 (5.0/92.0)
SF36.vital	*n =* 116: 41.0 ± 21.6 (0.0/90.0)	*n =* 146: 43.0 ± 20.3 (0.0/90.0)
SF36.social	67.4 ± 27.6 (0.0/100.0)	*n =* 146: 64.2 ± 27.9 (0.0/100.0)
SF36.rolem	*n =* 114: 67.5 ± 41.9 (0.0/100.0)	*n =* 146: 54.1 ± 44.0 (0.0/100.0)
SF36.mhi	*n =* 116: 62.5 ± 20.4 (16.0/96.0)	*n =* 146: 61.4 ± 21.4 (12.0/100.0)

^*^if not otherwise indicated, the full data set was used. Continuous variables are shown with mean ± SD and with min/max values in parenthesis.

ABC, Activities-specific Balance Confidence Scale; DHI, Dizziness Handicap Inventory; HADS-A, Hospital Anxiety and Depression Scale – Anxiety; HADS-D, Hospital Anxiety and Depression Scale – Depression; NPQ, Original Niigata PPPD Questionnaire (12 items); NPQ-R, Niigata PPPD Questionnaire Revised version (19 items); SF36.ghp, general health perceptions; SF36.mhi, general mental health; SF36.pain, physical pain; SF36.pfi, physical functioning; SF36.rolem, role limitations due to emotional problems; SF36.rolph, role limitations due to physical problems; SF36.soc, social functioning; SF36.vital, vitality; VSS, Vertigo Symptom Scale; VSS-A, Vertigo Symptom Scale – Anxiety; VSS-V, Vertigo Symptom Scale – Vertigo.

To determine test-retest reliability, 131 patients completed the NPQ-R twice. Mean age for the total population was 50.2 +/- 16.8 years (*M*_*female*_= 53.1 ± 15.8; *M*_*male*_= 47.09 ± 17.2). Disease duration was 46.3 ± 76.6 months (*M*_*female*_= 47.7 ± 77.4; *M*_*male*_= 44.8 ± 76.1) and therapy duration varied between zero and 144 months. Descriptive data for all questionnaires of the total study population is provided in Table 2. No forms had missing data exceeding the predefined 15%-level. The scores from the NPQ-R ranged from 4 to 100 points. Accordingly, no floor or ceiling effects were present (36).

Out of 118 patients in Switzerland, 114 returned the additional evaluation sheet regarding the NPQ-R's comprehensiveness and comprehensibility. Nearly all patients rated comprehensiveness and comprehensibility positively, with scores ranging between 92% and 99% for each of the 19 items.

Among the 148 PPPD patients in Germany, 95 patients were classified as having pPPPD and 53 as having sPPPD (16benign paroxysmal vertigo, 16 vestibular migraine, 8 acute unilateral vestibulopathy, 4 circulatory dysregulations, e.g., orthostatic, syncope, 3 infectious symptoms, 2 transient ischemic attack, 1 acceleration trauma). Statistical analyses revealed no significant differences neither of NPQ nor of NPQ-R in pPPPD vs. sPPPD (NPQ: pPPPD 29.1 ± 12.9 vs. sPPPD 30.1 ± 13.6, *p* = 0.675; NPQ-R: pPPPD 51.8 ±18.6 vs. sPPPD 52.8 ± 20.7, *p* = 0.776).

Table 2 presents an overview on all descriptive parameters for both sexes, indicating slightly higher mean values for females compared to males, with the exception of the SF-36 subscale for physical functioning and SF-36 subscale for role limitations due to physical problems.

Table 3 provides an overview of the correlations between the NPQ-R, the NPQ and all other patient-reported outcome measures utilized in the study. As expected, the correlation between the NPQ and NPQ-R was high (r_s_ = 0.969). The highest correlation for the NPQ and NPQ-R and all other patient-reported outcome measures was found between the NPQ-R and DHI (r_s_ = 0.752). VSS exhibit moderate (VSS: r_s_ = 0.498; VSS-V: r_s_ = 0.462; VSS-A: r_s_ = 0.430) correlations with NPQ-R and also moderate (VSS: r_s_ = 0.459; VSS-V: r_s_ = 0.426; VSS-A: r_s_ = 0.390) correlations with NPQ. Negative moderate correlations were found between NPQ-R and SF36 subscale physical functioning (r = −0.538) as well as NPQ (r = −0.578). HADS-A and HADS-D show moderate correlations with NPQ-R (r = 0.430 and 0.487) and NPQ (r = 0.354 and 0.438). ABC has a moderate negative correlation with both NPQ- R (r = −0.545) and NPQ (r = −0.542).

**Table 3:** Spearman rank correlations between NPQ-R, NPQ-R-Retest, NPQ and all other assessments (*n* = 131).

	**NPQ-R**	**NPQ-R-Retest**	**NPQ**	**DHI**	**VSS**	**VSS-V**	**VSS-A**	**ABC**	**HADS-A**	**HADS-D**
NPQ-R	-									
NPQ-R-retest	0.831	-								
	< 0.001^***^									
NPQ	0.969	0.813	-							
	< 0.001^***^	< 0.001^***^								
DHI	0.752	0.728	0.712	-						
	< 0.001^***^	< 0.001^***^	< 0.001^***^							
VSS	0.498	0.547	0.459	0.552	-					
	< 0.001^***^	< 0.001^***^	< 0.001^***^	< 0.001^***^						
VSS-V	0.462	0.518	0.426	0.498	0.909	-				
	< 0.001^***^	< 0.001^***^	< 0.001^***^	< 0.001^***^	< 0.001^***^					
VSS-A	0.430	0.469	0.390	0.494	0.856	0.589	-			
	< 0.001^***^	< 0.001^***^	< 0.001^***^	< 0.001^***^	< 0.001^***^	< 0.001^***^				
ABC	−0.545	−0.500	−0.542	−0.625	−0.284	−0.248	−0.269	-		
	< 0.001^***^	< 0.001^***^	< 0.001^***^	< 0.001^***^	< 0.001^***^	< 0.001^***^	< 0.001^***^			
HADS-A	0.430	0.387	0.354	0.464	0.540	0.417	0.585	−0.190	-	
	< 0.001^***^	< 0.001^***^	< 0.001^***^	< 0.001^***^	< 0.001^***^	< 0.001^***^	< 0.001^***^	0.002^**^		
HADS-D	0.487	0.457	0.438	0.600	0.418	0.345	0.411	−0.360	0.628	-
	< 0.001^***^	< 0.001^***^	< 0.001^***^	< 0.001^***^	< 0.001^***^	< 0.001^***^	< 0.001^***^	< 0.001^***^	< 0.001^***^	
SF36.pfi	−0.538	−0.534	−0.578	−0.656	−0.350	−0.299	−0.303	0.660	−0.157	−0.410
	< 0.001^***^	< 0.001^***^	< 0.001^***^	< 0.001^***^	< 0.001^***^	< 0.001^***^	< 0.001^***^	< 0.001^***^	< 0.011^*^	< 0.001^***^
SF36.rolph	−0.415	−0.388	−0.412	−0.533	−0.365	−0.319	−0.301	0.390	−0.265	−0.399
	< 0.001^***^	< 0.001^***^	< 0.001^***^	< 0.001^***^	< 0.001^***^	< 0.001^***^	< 0.001^***^	< 0.001^***^	< 0.001^***^	< 0.001^***^
SF36.pain	−0.318	−0.220	−0.316	−0.422	−0.383	−0.231	−0.506	0.336	−0.287	−0.281
	< 0.001^***^	0.013^*^	< 0.001^***^	< 0.001^***^	< 0.001^***^	< 0.001^***^	< 0.001^***^	< 0.001^***^	< 0.001^***^	< 0.001^***^
SF36.ghp	−0.372	−0.349	−0.360	−0.453	−0.456	−0.347	−0.481	0.301	−0.481	−0.527
	< 0.001^***^	< 0.001^***^	< 0.001^***^	< 0.001^***^	< 0.001^***^	< 0.001^***^	< 0.001^***^	< 0.001^***^	< 0.001^***^	< 0.001^***^
SF36.vital	−0.433	−0.439	−0.405	−0.506	−0.500	−0.385	−0.530	0.302	−0.495	−0.628
	< 0.001^***^	< 0.001^***^	< 0.001^***^	< 0.001^***^	< 0.001^***^	< 0.001^***^	< 0.001^***^	< 0.001^***^	< 0.001^***^	< 0.001^***^
SF36.social	−0.520	−0.457	−0.483	−0.623	−0.473	−0.409	−0.433	0.347	−0.497	−0.619
	< 0.001^***^	< 0.001^***^	< 0.001^***^	< 0.001^***^	< 0.001^***^	< 0.001^***^	< 0.001^***^	< 0.001^***^	< 0.001^***^	< 0.001^***^
SF36.rolem	−0.251	–	−0.216	−0.325	−0.293	−0.275	−0.259	0.181	−0.519	−0.476
	< 0.001^***^	0.1440.104	< 0.001^***^	< 0.001^***^	< 0.001^***^	< 0.001^***^	< 0.001^***^	0.003^**^	< 0.001^***^	< 0.001^***^
SF36.mhi	−0.441	−0.420	−0.389	−0.491	−0.438	−0.366	−0.434	0.252	−0.722	−0.714
	< 0.001^***^	< 0.001^***^	< 0.001^***^	< 0.001^***^	< 0.001^***^	< 0.001^***^	< 0.001^***^	< 0.001^***^	< 0.001^***^	< 0.001^***^

ABC, Activities-specific Balance Confidence Scale; DHI, Dizziness Handicap Inventory; HADS-A, Hospital Anxiety and Depression Scale–Anxiety; HADS-D, Hospital Anxiety and Depression Scale–Depression; NPQ, Original Niigata PPPD Questionnaire (12 items); NPQ-R, Niigata PPPD Questionnaire Revised version (19 items); SF36.ghp, general health perceptions; SF36.mhi, general mental health; SF36.pain, physical pain; SF36.pfi, physical functioning; SF36.rolem, role limitations due to emotional problems; SF36.rolph, role limitations due to physical problems; SF36.soc, social functioning; SF36.vital, vitality; VSS, Vertigo Symptom Scale; VSS-A, Vertigo Symptom Scale–Anxiety; VSS-V, Vertigo Symptom Scale–Vertigo. ^*^, ^**^, ^***^ Significance level of *p* < 0.05/0.01/0.001.

### 3.2 Reliability and reliability-related parameters: internal consistency, test-retest reliability, SEM, and MDC

#### 3.2.1 Internal consistency

Cronbach's α values ranged between 0.62 and 0.78 for the subscales separately and α=0.91 for the 19-item NPQ-R (Table 4). For comparison purposes, the values of the 12-item NPQ version are also shown in Table 5.

**Table 4:** Descriptive analysis for the total study population and for both sexes.

	**Total (*n* = 265)**	**Males (*n* = 130)**	**Females (*n* = 135)**
Age	*n* = 265: 50.2 ± 16.8 (17.0/83.0)	*n* = 130: 47.1 ± 17.2 (17.0/81.0)	*n* = 135: 53.1 ± 15.8 (18.0/83.0)
Duration of dizziness [months]	*n* = 265: 46.3 ± 76.6 (1.0/432.0; *n* = 250)	*n* = 121: 44.8 ± 76.1 (1.0/432.0)	*n* = 129: 47.7 ± 77.4 (1.0/432.0)
Duration of therapy [months]	*n* = 233: 5.2 ± 13.3 (0.0/144.0)	*n* = 116: 3.5 ± 8.6 (0.0/60.0)	*n* = 117: **6.8** ± 16.5 (0.0/144.0)
Sport/Week [frequency per week]	*n* = 256: 2.4 ± 2.2 (0.0/7.0)	*n* = 125: 2.6 ± 2.3 (0.0/7.0)	*n* = 131: 2.3 ± 2.1 (0.0/7.0)
NPQ-R (19 items)	*n* = 265: 52.3 ± 19.6 (4.0/100.0)	*n* = 130: 48.2 ± 19.3 (4.0/93.0)	*n* = 135: **56.3** ± 19.1 (10.0/100.0)
NPQ-R-Retest	*n* = 131: 48.6 ± 21.1 (2.0/102.0)	*n* = 59: 43.5 ± 21.4 (2.0/84.0)	*n* = 72: **52.8** ± 19.8 (9.0/102.0)
NPQ (12 items)	*n* = 265: 29.9 ± 13.2 (0.0/63.0)	*n* = 130: 27.0 ± 13.0 (0.0/59.0)	*n* = 135: **32.6** ± 12.9 (3.0/63.0)
DHI	*n* = 265: 46.1 ± 19.6 (0.0/88.0)	*n* = 130: 43.2 ± 19.1 (0.0/84.0)	*n* = 135: **49.0** ± 19.8 (0.0/88.0)
VSS-Total	*n* = 265: 42.6 ± 23.2 (0.0/113.0)	*n* = 130: 42.0 ± 23.0 (4.0/113.0)	*n* = 135: 43.1 ± 23.1 (0.0/101.0)
VSS-V	*n* = 265: 21.1 ± 14.7 (0.0/65.0)	*n* = 130: 20.4 ± 14.5 (0.0/65.0)	*n* = 135: 21.7 ± 14.9 (0.0/61.0)
VSS-A	*n* = 265: 21.5 ± 11.7 (0.0/51.0)	*n* = 130: 21.6 ± 11.7 (1.0/51.0)	*n* = 135: 21.4 ± 11.7 (0.0/51.0)
ABC	*n* = 264: 0.7 ± 0.2 (0.0/1.0)	*n* = 130: 0.8 ± 0.2 (0.01/1.0)	*n* = 134: 0.7 ± 0.2 (0.05/1.0)
HADS-A	*n* = 264: 8.0 ± 4.4 (0.0/20.0)	*n* = 130: 8.2 ± 4.5 (0.0/17.0)	*n* = 134: 7.9 ± 4.3 (0.0/20.0)
HADS-D	*n* = 264: 6.5 ± 4.1 (0.0/19.0)	*n* = 130: 7.0 ± 4.3 (0.0/19.0)	*n* = 134: **6.1** ± 3.8 (0.0/18.0)
SF36.pfi	*n* = 263: 69.3 ± 25.0 (0.0/100.0)	*n* = 130: **73.9** ± 24.3 (0.0/100.0)	*n* = 133: 64.8 ± 24.9 (0.0/100.0)
SF36.rolph	*n* = 260: 42.8 ± 41.7 (0.0/100.0)	n =129: **45.0** ± 43.1 (0.0/100.0)	*n* = 131: 40.7 ± 40.4 (0.0/100.0)
SF36.pain	*n* = 260: 63.5 ± 28.5 (0.0/100.0)	*n* = 129: 63.2 ± 29.1 (0.0/100.0)	*n* = 131: 63.8 ± 28.1 (0.0/100.0)
SF36.ghp	*n* = 260: 52.8 ± 20.4 (5.0/97.0)	*n* = 128: 51.1 ± 20.8 (5.0/96.25)	*n* = 132: 54.6 ± 19.9 (5.0/97.0)
SF36.vital	*n* = 262: 42.1 ± 20.9 (0.0/90.0)	*n* = 129: 42.5 ± 21.7 (0.0/90.0)	*n* = 133: 41.8 ± 20.1 (0.0/90.0)
SF36.social	*n* = 263: 65.6 ± 27.8 (0.0/100.0)	*n* = 130: 66.8 ± 27.7 (0.0/100.0)	*n* = 133: 64.5 ± 27.9 (0.0/100.0)
SF36.rolem	*n* = 260: 60.0 ± 43.5 (0.0/100.0)	*n* = 129: 56.9 ± 44.0 (0.0/100.0)	*n* = 131: **63.1** ± 43.0 (0.0/100.0)
SF36.mhi	*n* = 262: 61.9 ± 20.9 (12.0/100.0)	*n* = 129: 61.5 ± 21.7 (16.0/96.0)	*n* = 133: 62.3 ± 20.3 (12.0/100.0)

Legend: Numbers in bold indicate slightly higher mean values for females compared to males except for SF-36 physical functioning. Continuous variables are shown with mean ± SD and with min/max values in parenthesis.

ABC, Activities-specific Balance Confidence Scale; DHI, Dizziness Handicap Inventory; HADS-A, Hospital Anxiety and Depression Scale – Anxiety; HADS-D, Hospital Anxiety and Depression Scale – Depression; NPQ, Original Niigata PPPD Questionnaire (12 items); NPQ-R, Niigata PPPD Questionnaire Revised version (19 items); SF36.ghp, general health perceptions; SF36.mhi, general mental health; SF36.pain, physical pain; SF36.pfi, physical functioning; SF36.rolem, role limitations due to emotional problems; SF36.rolph, role limitations due to physical problems; SF36.soc, social functioning; SF36.vital, vitality; VSS, Vertigo Symptom Scale; VSS-A, Vertigo Symptom Scale – Anxiety; VSS-V, Vertigo Symptom Scale – Vertigo.

**Table 5:** Cronbach's alpha for NPQ-R and NPQ (n = 265).

	**Total NPQ (12 items)**	**Total NPQ-R (19 items)**	**Subscale upright posture/ walking**	**Subscale movement**	**Subscale visual**	**Subscale associated symptoms; NPQ-R**	**Subscale symptom behavior; NPQ-R**
Cronbach's alpha (95% CI)	0.88 (0.86–0.90)	0.91 (0.90–0.93)	0.76 (0.71–0.80)	0.73 (0.67–0.78)	0.78 (0.73–0.82)	0.71 (0.65–0.76)	0.62 (0.53–0.69)

NPQ, Niigata PPPD Questionnaire; NPQ-R, Niigata PPPD Questionnaire Revised; CI, Confidence interval.

Furthermore, item analyses for the subscales revealed a higher α in each subscale, if the following items within one subscale were dropped: upright postured/walking item 11 (α = 0.734), movement item 8 (α = 0.717), visual item 2 (α = 0.799), associated symptoms (NPQ-R) item 9 (α = 0.738), and symptom behavior (NPQ-R) item 7 (α = 0.560) as seen in Table 6.

**Table 6:** Item analysis of the NPQ-R when dropping one item per subscale (n = 265).

**Dropped item**	**Cronbach's alpha NPQ-R when dropping a item of the subscale**	**Item mean**	**Item SD**
**Upright posture**			
NPQ-R 4	0.695	2.540	1.448
NPQ-R 11	0.734	2.189	1.673
NPQ-R 12	0.684	2.955	1.780
NPQ-R 18	0.698	2.644	1.556
**Movement**			
NPQ-R 1	0.680	2.886	1.556
NPQ-R 8	0.717	2.328	1.666
NPQ-R 15	0.647	2.551	1.487
NPQ-R 19	0.637	2.498	1.706
**Visual**			
NPQ-R 2	0.799	2.650	1.712
NPQ-R 6	0.720	2.389	1.932
NPQ-R 13	0.668	2.466	1.793
NPQ-R 16	0.708	1.867	1.632
**Associated symptoms**			
NPQ-R 3	0.631	4.221	1.586
NPQ-R 5	0.584	3.780	1.799
NPQ-R 9	0.738	2.774	1.801
NPQ-R 17	0.618	4.455	1.554
**Symptom behavior**			
NPQ-R 7	0.560	1.728	1.407
NPQ-R 10	0.526	3.502	1.697
NPQ-R 14	0.468	2.073	1.336

NPQ-R, Niigata PPPD Questionnaire revised version.

#### 3.2.2 Test-retest reliability

Test-retest reliability scores of the NPQ and the NPQ-R including the **SEM** and the **MDC** values are provided in Table 7 and in the Bland Altman Plot in Figure 3. The second NPQ-R was only sent after the first one had been received. In the meantime, patients were reminded to complete the first questionnaire as soon as possible to minimize the time gap between the two assessments. Thus, the NPQ-R was then sent for the retest between 7 and 99 days afterwards (median = 9 days; IQR = 14 days). The central line in the Bland Altman plot shows the mean difference (MD = 2.5) in NPQ-R total score between the first and second measurement and its limits of agreement (95% of the differences between measurement time point one and two are between −20.1 and 25.1) (37).

**Table 7:** ICC, SEM, MDC, mean difference and range of agreement for the NPQ (*n* = 131) and NPQ-R (*n* = 131).

	**ICC (2.1)**	**SEM**	**MDC**	**Mean difference**	**Range of agreement**
NPQ (12 items)	0.83 (CI 0.79 to 0.87)	5.55	15 points	1.14	−13.78–16.05
NPQ-R (19 items)	0.83 (CI 0.77 to 0.86)	8.37	23 points	2.52	−20.09–25.13

ICC, Intraclass correlation; NPQ, Niigata PPPD questionnaire; NPQ-R, Niigata PPPD questionnaire revised; SEM, Standard error of the mean; MDC, Minimal detectable change.

## 4 Discussion

Persistent postural-perceptual Dizziness (PPPD) is defined as a chronic vestibular disorder of non-spinning vertigo that is exacerbated by three factors: upright posture, active or passive movements, and exposure to moving or complex visual patterns. It causes significant distress as well as functional impairment (1). The diagnosis of this functional neurological disorder is based on the presence of clinical key symptoms as positive characteristics as well as the exclusion of another disease. Due to the lack of a specific biomarker, the diagnosis of PPPD requires a precise assessment of vestibular symptoms, exacerbating factors, and the medical history. The Niigata PPPD Questionnaire (NPQ), designed for the standardized diagnosis and assessment of the severity of PPPD, was first developed in Japan in 2019 (8). The original NPQ was recently translated into a German version and revised through an expert Delphi consensus survey, incorporating additional items that address symptoms and symptom behavior to provide a comprehensive assessment of PPPD intensity (NPQ-R) (11). The present study assessed the convergent construct validity, internal consistency, test-retest reliability, SEM and MDC of both the translated German 12-item original NPQ and the 19-item revised NPQ-R version. Our results indicate that both versions are valid and reliable patient-reported outcome measures for assessing and monitoring symptomatology in patients with persistent postural perceptual dizziness. However, we recommend the 19-item NPQ-R due to its incorporation of both patient and expert perspectives, as well as its higher internal consistency of the total score compared to the 12-item NPQ.

### 4.1 Convergent construct validity

Overall, our hypothesis concerning construct validity was confirmed. Convergent construct validity of the NPQ and NPQ-R was established through the strong association between the NPQ and NPQ-R with the DHI (r_s_ = 0.712; r_s_ = 0.752). The high correlations suggest that the DHI captures similar triggers and functional limitations, indicating that it includes items related to the construct under investigation and is comparable to the Spanish version with r_P_=0.751 (36) and the French version with 0.73 (38).

The weak to moderate correlations of the NPQ and NPQ-R with the anxiety subscales VSS-A (r_s_ = 0.390; r_s_ = 0.430) and HADS-A (r_s_ = 0.354; r_s_ = 0.430) as well as with the depression subscale of the HADS (r_s_ = 0.438 and r_s_ = 0.487) suggest that the association between PPPD and psychiatric illness might be low, which corresponds to the French cohort with 50 patients with r_s_ = 0.27 for HADS-D, 0.08 for HADS-A, and 0.07 for anxiety. These values are line with earlier studies in precursor functional vertigo syndromes, e.g., chronic subjective dizziness or somatoform vertigo (20, 39) as well as with the smaller French and Spanish evaluation (HADS-D 8.5, HADS-A 11.5). However, PPPD is considered a specific functional diagnosis rather than a psychiatric diagnosis (16, 18).

The ABC-score reflects the patients' confidence in performing certain activities. In our study, the ABC-score indicated only moderately impaired level of functioning (mean 0.7, Table 4), and the negative correlation between the NPQ/NPQ-R and the ABC-Scale (r_s_ = −0.545) was even moderate. This fits to the current pathophysiological concept of PPPD that postulates an increased self-observation with a shift to high-demand postural control strategies (stiffening due to higher anti-gravity muscle activity and co-contraction). These changes can be identified by posturography and gait analyses (18, 40–43): In functional dizziness the postural sway is typically increased during simple balance tasks, but becomes normal with distraction (dual tasks) and more difficult balance tasks, i.e., the more difficult the demand of balance, the more “healthy” is the balance performance. In this context, and consistent with typical patients' reports indicating an improvement in symptoms with physical activities, it seems logical that the overall confidence of patients in performing activities is only slightly diminished. The finding of a less strong calculated association between ABC and NPQ in our study compared to the Spanish study (r_p_= −0.739) is due to their inhomogeneous patient cohort including not only PPPD, but two third of patients with organic vestibular syndromes and probable persisting vestibular impairment (36, 40–43).

Only low to moderate negative correlations were observed between the NPQ and NPQ-R and the SF-36 subscales, supporting our hypothesis that the SF-36, as a generic measure of overall quality of life, and the NPQ, as a disease-specific construct measure, assess different parameters. However, the negative correlations indicate that PPPD adversely affects health-related quality of life, impacting both physical and social functioning, thereby underscoring its symptomatic impact and relevance in daily life. No statistically significant differences were found between the 12-item NPQ and the 19-item NPQ-R regarding the associations between the disease-, depression-, anxiety- or general health-related questionnaires.

### 4.2 Reliability and reliability-related parameters: internal consistency, test-retest reliability, SEM, and MDC

The **Internal consistency** of the NPQ and NPQ-R can be considered highly acceptable (alpha = 0.88; alpha = 0.91). Subscale analyses also revealed acceptable values ranging between 0.71 and 0.78. Our alpha values are lower compared to the French (38) and the Spanish (36) NPQ versions with alpha values ranging between 0.81 and 0.92 and between 0.803 and 0.869, respectively. However, authors of both studies included distinct smaller patient cohorts of only 50 and 47 PPPD patients. The new subscale for symptom behavior of the NPQ-R demonstrated a moderate internal consistency with α=0.62, which falls below the recommended threshold of Tavakol and Dennick (30). These authors suggested that a lower level of internal consistency may be attributed to a small number of items within the subscale, which is the case with three items compared to four items in all other subscales. However, the symptom behavior subscale still provides information regarding the change in PPPD symptoms over time or the patient's ability to adapt to the limiting condition. Therefore, we recommend using the NPQ-R with all subscales rather than individual subscales only.

**Figure 2:**
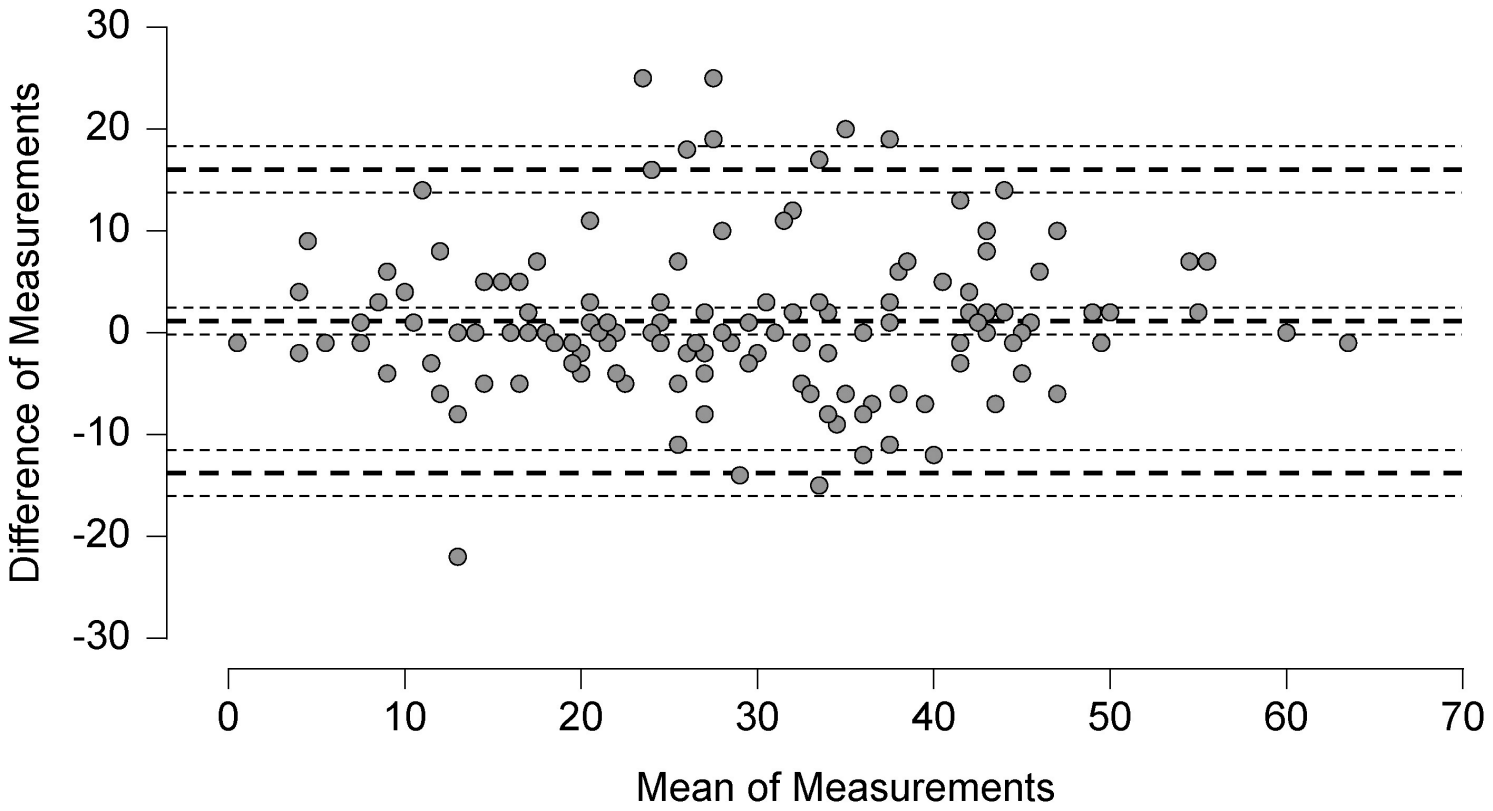
Bland altman plot: analysis of differences between the NPQ scores at test and retest.

**Figure 3:**
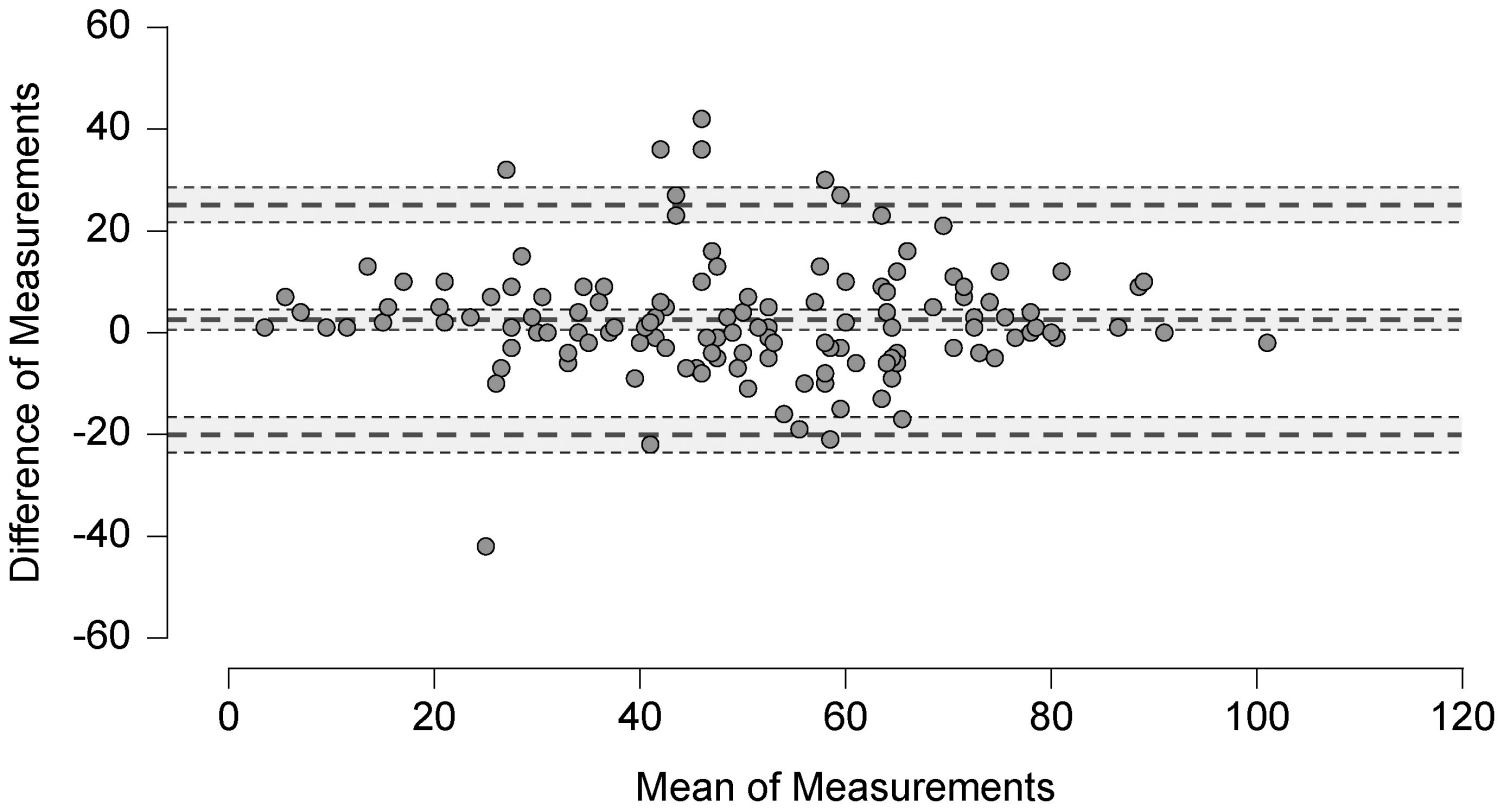
Bland altman plot: analysis of differences between the NPQ-R scores at test and retest.

**Test-retest reliability** for the NPQ (Figure 2) and the NPQ-R (Figure 3) appears to be satisfactory and is comparable to the ICC scores reported for the Spanish NPQ version (36).

Due to our large sample size with *n* = 265, the SEM (5.53) of the NPQ (12 items) is lower than the **SEM** the Spanish version (6.63) (36), based on 47 patients with PPPD only. Their smaller sample size might be the reason for their lower MDC with 12.99 compared to our MDC with 15.5 points for the NPQ and with 23 points for the NPQ-R.

Both Yagi et al. (8) for the original Japanese and Meletaki et al. (38) for the French version did not provide information regarding test-retest reliability, SEM and MDC.

### 4.3 Outlook

Our inter-item analysis indicated promising values for the differentiation ability of the NPQ-R from a descriptive perspective. All items except items 2 (“*When I look through shelves in the supermarket or hardware store”)* and 9 (“*When I walk, I feel insecure*”) demonstrated good internal consistency. Obviously, item 2 and 9 are not very specific for PPPD, since walking or standing can be influenced by several other stance and gait relevant limitations, e.g., polyneuropathy, musculoskeletal disorders, vision problems etc.

For the German NPQ and NPQ-R, we did not assess one-dimensionality and equivalence, which would extent the construct validity of the NPQ and NPQ-R. Therefore, an exploratory and confirmatory factor analyses are warranted.

### 4.4 Strengths and limitations

Following the COSMIN recommendations, we present the largest sample size (*n* = 265) compared to the original Japanese (*n* = 50), the French (n = 50), and the Spanish (*n* = 47) versions. This substantial sample size provides a robust foundation for the investigation of the psychometric properties of the NPQ and NPQ-R, thereby supporting their application in clinical practice. Furthermore, we provide data on the duration of the PPPD symptoms, the patients' therapy, and their weekly physical activity levels. Additionally, a comprehensive investigation of the 12-item NPQ and 19-item NPQ-R was conducted, examining their correlations with questionnaires related to anxiety, depression, and general health.

Strength of the study, in addition to the large number of patients included, was the recruitment of participants from different outpatient and inpatient sectors across different health care systems in two German-speaking countries. This included private practices, local, regional and university hospitals, which underscores the good applicability of the NPQ and NPQ-R. We would like to emphasize, that there were no statistical differences in the validation of the NPQ and NPQ-R between the German and Swiss cohorts, making a selection bias unlikely. For a more detailed interpretation of the associations between NPQ/NPQ- R and HADS-A and HADS-D, a more comprehensive psychiatric assessment of psychiatric comorbidities would have been beneficial. However, conducting such structured clinical interviews by a psychologist or psychiatrist would have been resource-intensive and fell outside the primary scope of this study. Nonetheless, the prevalence of depressive and anxiety symptoms in our patient cohort is in line with earlier reports (20, 39).

In our study, we focused extensively on the convergent construct validity and reliability parameters of the translated NPQ and the revised NPQ-R. Compared to Yagi et al. (8), Meletaki et al. (38), and Castillejos-Carrasco-Muñoz et al. (36), we were not interested in the discriminant validity of the NPQ, and NPQ- R. To assess the discriminative property for the German versions, a comparative investigation involving patients with PPPD alongside a sufficiently large sample of patients with various vestibular disorders and concurrent diverse symptomatology will be necessary in the future.

A minor limitation of this study could be the inclusion of patients who had already been informed of their diagnosis and received treatment, alongside treatment-naive patients. Combining these two groups may skew results, as both awareness of the diagnosis and prior therapy could significantly influence subjective PPPD symptoms.

The variable duration of the test-retest reliability period may also be regarded as an undesirable influencing factor. Some participants required more days to return the second NPQ-R questionnaire. However, we verified that the average scores for the NPQ and NPQ-R did not differ from those obtained during the first measurement event and are also comparable to the scores from the Japanese, French, and Spanish versions. Furthermore, the mean symptom duration at first measurement was 46 months (Table 4), and a relevant change of the symptomatology of such a chronic disease within a few days appears unlikely. Additionally, we performed a subgroup analysis to gain insights into a potential correlation between interval length and test-retest reliability score. For the NPQ-R data, we divided the whole study population in to a group with a 7 days test-retest duration (*n* = 40; ICC2, 1 = 0.913), a group with a 8 to 30 days test-retest duration (*n* = 66; ICC2,1 = 0.832), and a 31+ days test-retest duration (*n* = 9; ICC2,1 = 0.853). The results did not indicate a relevant impact on the overall test-retest scores for the study participants.

## 5 Conclusions

The total scale of the German version of the Niigata PPPD Questionnaire and its revised version both demonstrated satisfactory measurement properties for convergent construct validity and reliability parameters (internal consistency, test-retest, SEM and MDC) as an evaluative measure. They represent valid and reliable patient-reported outcome measures suitable for routine clinical use across various health care settings. Compared to the NPQ (12 items), the NPQ-R with its 19 items appears to capture the clinical course of PPPD equally well. However, we recommend the NPQ-R version for the evaluation of PPPD treatment, as it further considers symptom behavior and associated symptoms from the patient's and clinician's perspectives, contributing significantly to the standardized assessment of health status. The German NPQ and NPQ-R should be further evaluated for their multidimensionality through factor analytic analyses (structure-seeking and structure-confirming) and for their ability to discriminate other organic vestibular syndromes.

## Data Availability

The raw data supporting the conclusions of this article will be made available by the authors, without undue reservation.
