# Supplementary material for: Convergent construct validity and test-retest reliability of both German versions of the original and the revised Niigata PPPD Questionnaire: NPQ and NPQ-R
Source: Front Neurol. 2025 Jan 27;16:1517566. doi: 10.3389/fneur.2025.1517566 (PMC11807810; doi:10.3389/fneur.2025.1517566)
Supplement: Supplementary file 1 [file Data_Sheet_1.pdf]

The Niigata PPPD Questionnaire Revised (NPQ-R, 12 items)

Original: Universität Niigata, HNO-Abteilung: Yagi, C., Y. Morita, M. Kitazawa, Y. Nonomura, T. Yamagishi, S. Ohshima, S. Izumi, K. Takahashi and A. Horii (2019). "A Validated Questionnaire to Assess the Severity of Persistent Postural-Perceptual Dizziness (PPPD): The Niigata PPPD Questionnaire (NPQ)." Otol Neurotol **40**(7): e747-e752.

Name / Number Patient: .... Date: ....

This questionnaire is designed to help you better understand your dizziness / lightheadedness symptoms. Please rate the severity of your discomfort using the questions below on a 7-point scale from 0 to 6. Please circle the applicable answer.
If you completely avoid the actions mentioned in a question so as not to increase your discomfort, please circle the number 6 ("unbearable").

If your complaints are not always the same, please evaluate them based on the most severe occurrence during the last 7 days.
This questionnaire has three pages.

Example 1:

I have no complaints Is it unbearable.

0 1 (2) 3 4 5 6

1. When I stand up quickly, turn around quickly, or in similar movements, then

   I have no complaints Is it unbearable.

   0 1 2 3 4 5 6

2. When I look through shelves in the supermarket or hardware store, then

   I have no complaints Is it unbearable.

   0 1 2 3 4 5 6

3. When I walk at my own pace, then

   I have no complaints Is it unbearable.

   0 1 2 3 4 5 6

Please continue on page 2 ➔

Copying permitted - Please do not modify.
Translation of the original by Reha Rheinfelden, Switzerland
Contakt: c.schuster@reha-rhf.ch

4. When I see fast/hectic images in movies or on TV, then
I have no complaints Is it unbearable.
0 1 2 3 4 5 6

5. When I travel by car, bus, train or other means of transport, then
I have no complaints Is it unbearable.
0 1 2 3 4 5 6

6. If I sit for a longer period of time on a stool or a chair without back or armrests, then
I have no complaints Is it unbearable.
0 1 2 3 4 5 6

7. If I stand freely for a longer period of time without holding on or leaning up, then
I have no complaints Is it unbearable.
0 1 2 3 4 5 6

8. When I scroll through the screen contents on a PC or smartphone, then
I have no complaints Is it unbearable.
0 1 2 3 4 5 6

9. When I am doing household chores or light sports, then
I have no complaints Is it unbearable.
0 1 2 3 4 5 6

10. When I read small print in books or the newspaper, then
I have no complaints Is it unbearable.
0 1 2 3 4 5 6

Please continue on page 3 ➔

Copying permitted - Please do not modify.
Translation of the original by Reha Rheinfelden, Switzerland
Contakt: c.schuster@reha-rhf.ch

11. When I walk with big steps and rather fast, then

I have no complaints								Is it unbearable.
	0	1	2	3	4	5	6	

12. When I use escalators or an elevator, then

I have no complaints								Is it unbearable.
	0	1	2	3	4	5	6	

Thank you very much for your cooperation.

Please do not enter anything here.

Upright posture / Standing (= Items 4+11+12+18)	Points
While moving (= Items 1+8+15+19)	Points
Visual (= Items 2+6+13+16)	Points
**Associated symptoms (= Items 3+5+9+17)**	Points
**Symptom behaviour (= Items 7+10+14)**	Points
Total	Points

Copying permitted - Please do not modify.
Translation of the original by Reha Rheinfelden, Switzerland
Contakt: c.schuster@reha-rhf.ch
