# Supplementary material for: Convergent construct validity and test-retest reliability of both German versions of the original and the revised Niigata PPPD Questionnaire: NPQ and NPQ-R
Source: Front Neurol. 2025 Jan 27;16:1517566. doi: 10.3389/fneur.2025.1517566 (PMC11807810; doi:10.3389/fneur.2025.1517566)
Supplement: Supplementary file 3 [file Data_Sheet_3.pdf]

Fragebogen zu chronischen Schwindelsymptomen
Revidierte Version des Niigata PPPD-Fragebogens (NPQ-R, 19 Items)

Original: Universität Niigata, HNO-Abteilung: Yagi, C., Y. Morita, M. Kitazawa, Y. Nonomura, T. Yamagishi, S. Ohshima, S. Izumi, K. Takahashi and A. Horii (2019). "A Validated Questionnaire to Assess the Severity of Persistent Postural-Perceptual Dizziness (PPPD): The Niigata PPPD Questionnaire (NPQ)." Otol Neurotol **40**(7): e747-e752.

Name / Nummer Patient: .... Datum: ....

Dieser Fragebogen dient dazu, Ihre Schwindel- / Benommenheitsbeschwerden besser zu erfassen. Bitte bewerten Sie die Stärke Ihrer Beschwerden anhand der untenstehenden Fragen auf einer 7-stufigen Skala von 0 bis 6. Bitte kreisen Sie die zutreffende Antwort ein.
Wenn Sie die in einer Frage genannten Handlungen komplett vermeiden, um Ihre Beschwerden nicht zu verstärken, umkreisen Sie bitte die Zahl 6 («nicht auszuhalten»).

Wenn Ihre Beschwerden nicht immer gleich sind, bewerten Sie sie bitte ausgehend vom stärksten Auftreten während der letzten 7 Tage.
Dieser Fragebogen hat vier Seiten.

Beispiel 1:

Habe ich keine Beschwerden — Ist es nicht auszuhalten

0 1 (2) 3 4 5 6

Beispiel 2:

Trifft überhaupt nicht zu — Trifft voll und ganz zu

0 1 2 (3) 4 5 6

1. Wenn ich schnell aufstehe, mich schnell umdrehe oder bei ähnlichen Bewegungen, dann
   habe ich keine Beschwerden — ist es nicht auszuhalten.
   0 1 2 3 4 5 6

2. Wenn ich Regale im Supermarkt oder Baumarkt durchsehe, dann
   habe ich keine Beschwerden — ist es nicht auszuhalten.
   0 1 2 3 4 5 6

Bitte weiter auf Seite 2 ➔

3. Wenn ich Schwindel habe, dann habe ich Mühe, mich zu konzentrieren.
   Trifft überhaupt nicht zu — Trifft voll und ganz zu.
   0 1 2 3 4 5 6

4. Wenn ich in meinem eigenen Tempo zu Fuss gehe, dann
   habe ich keine Beschwerden — ist es nicht auszuhalten.
   0 1 2 3 4 5 6

5. Wenn ich Schwindel habe, dann fühle ich mich verängstigt oder verunsichert.
   Trifft überhaupt nicht zu — Trifft voll und ganz zu.
   0 1 2 3 4 5 6

6. Wenn ich in Film oder Fernsehen schnelle/hektische Bilder sehe, dann
   habe ich keine Beschwerden — ist es nicht auszuhalten.
   0 1 2 3 4 5 6

7. Wenn ich mir eine Pause gönne oder mich ausruhe, dann
   habe ich keine Beschwerden — ist es nicht auszuhalten.
   0 1 2 3 4 5 6

8. Wenn ich mit Auto, Bus, Zug oder anderen Verkehrsmitteln fahre, dann
   habe ich keine Beschwerden — ist es nicht auszuhalten.
   0 1 2 3 4 5 6

9. Wenn ich zu Fuss gehe, dann fühle ich mich unsicher.
   Trifft überhaupt nicht zu — Trifft voll und ganz zu.
   0 1 2 3 4 5 6

10. Wenn ich mich in unruhiger Umgebung befinde (z.B. Menschenmenge, Verkehr), dann
   habe ich keine Beschwerden — ist es nicht auszuhalten.
   0 1 2 3 4 5 6

Bitte weiter auf Seite 3 ➔

Kopieren erlaubt – Bitte nicht verändern
Übersetzung des Originals durch Reha Rheinfelden, Schweiz
Kontakt: c.schuster@reha-rhf.ch

11. Wenn ich länger auf einem Hocker oder einem Stuhl ohne Rücken- oder Armlehnen sitze, dann
    habe ich keine Beschwerden ... ist es nicht auszuhalten.
    0 1 2 3 4 5 6

12. Wenn ich länger frei stehe ohne mich fest zu halten oder mich auf zu stützen, dann
    habe ich keine Beschwerden ... ist es nicht auszuhalten.
    0 1 2 3 4 5 6

13. Wenn ich auf einem PC oder Smartphone den Bildschirminhalt durchscrolle, dann
    habe ich keine Beschwerden ... ist es nicht auszuhalten.
    0 1 2 3 4 5 6

14. Wenn ich mich ablenke z.B. durch eine Tätigkeit oder meine Gedanken auf etwas anderes richte, dann
    habe ich keine Beschwerden ... ist es nicht auszuhalten.
    0 1 2 3 4 5 6

15. Wenn ich mich im Haushalt oder bei leichtem Sport bewege, dann
    habe ich keine Beschwerden ... ist es nicht auszuhalten.
    0 1 2 3 4 5 6

16. Wenn ich klein gedruckte Schrift in Büchern oder der Zeitung lese, dann
    habe ich keine Beschwerden ... ist es nicht auszuhalten.
    0 1 2 3 4 5 6

17. Wenn ich Schwindel habe, dann ist meine Leistungsfähigkeit eingeschränkt (z.B. Beruf, Kinderbetreuung, häusliche Tätigkeit).
    Trifft überhaupt nicht zu ... Trifft voll und ganz zu.
    0 1 2 3 4 5 6

Bitte weiter auf Seite 4 ➔

Kopieren erlaubt – Bitte nicht verändern
Übersetzung des Originals durch Reha Rheinfelden, Schweiz
Kontakt: c.schuster@reha-rhf.ch

18. Wenn ich mit grossen Schritten und eher schnell gehe, dann

habe ich keine Beschwerden ... ist es nicht auszuhalten.

0 1 2 3 4 5 6

19. Wenn ich Rolltreppen oder einen Aufzug benutze, dann

habe ich keine Beschwerden ... ist es nicht auszuhalten.

0 1 2 3 4 5 6

Herzlichen Dank für Ihre Mitarbeit.

Hier bitte nichts eintragen.

Aufrecht / Stehend (= Items 4+11+12+18)________Punkte

In Bewegung (= Items 1+8+15+19)________Punkte

Visuell (= Items 2+6+13+16)________Punkte

Begleitsymptome (= Items 3+5+9+17)________Punkte

Symptomverhalten (= Items 7+10+14)________Punkte

Gesamt________Punkte

Kopieren erlaubt – Bitte nicht verändern

Übersetzung des Originals durch Reha Rheinfelden, Schweiz

Kontakt: c.schuster@reha-rhf.ch
